# Evaluation of Aminated Nano-Silica as a Novel Shale Stabilizer to Improve Wellbore Stability

**DOI:** 10.3390/ma17081776

**Published:** 2024-04-12

**Authors:** Meng Li, Jiangen Xu, Dongdong Pei, Kanhua Su, Liang Wang

**Affiliations:** 1 School of Petroleum Engineering, Chongqing University of Science & Technology, Chongqing 401331, China; 2016019@cqust.edu.cn (M.L.);; 2 Safety and Environmental Protection Technology Supervision Center, PetroChina Liaohe Oilfield Company, Panjin 124010, China

**Keywords:** shale stabilizer, wellbore stability, dense plugging, inhibition

## Abstract

The issue of wellbore instability poses a significant challenge in the current exploration of shale gas reservoirs. Exploring more efficient shale stabilizers has always been a common goal pursued by researchers. In this paper, a novel shale stabilizer, denoted as ANS, was prepared by employing a silane-coupling modification method to graft (3-Aminopropyl) triethoxysilane (APTES) onto the surface of nano-silica. The structure of ANS was characterized through Fourier transforms infrared spectroscopy (FT-IR), thermo-gravimetric analysis (TGA), and particle size tests (PST). The shale stabilizing properties of ANS were evaluated through tests such as pressure penetration, BET analysis, hydration expansion and dispersion. Furthermore, the interaction between ANS as a shale stabilizer and clay was explored through clay zeta potential and particle size analysis. The results indicated that ANS exhibited a stronger plugging capability compared to nano-silica, as evidenced by its ability to increase the shale pressure penetration time from 19 to 131 min. Moreover, ANS demonstrated superior hydration inhibition compared to commonly used KCl. Specifically, it reduced the expansion height of bentonite from 8.04 to 3.13 mm and increased the shale recovery rate from 32.84% to 87.22%. Consequently, ANS played a dual role in providing dense plugging and effective hydration inhibition, contributing significantly to the enhancement of wellbore stability in drilling operations. Overall, ANS proved to be a promising shale stabilizer and could be effective for drilling troublesome shales.

## 1. Introduction

In recent years, the global economy has entered a new development cycle, leading to a sharp increase in the demand for petroleum and natural gas resources worldwide [1,2,3]. Confronted with substantial energy needs, there is a growing focus on unconventional oil and gas resources. Shale gas, as a clean unconventional resource, has become a focal point in exploration and development [4,5]. Shale formations contain abundant clay minerals, making them highly sensitive to water and prone to hydration [6,7]. The hydration process often leads to shale expansion and dispersion, resulting in issues such as wellbore instability, stuck pipe, bit balling, and mud losses [8,9,10]. Addressing these challenges often requires more time and financial resources for maintenance.

Oil-based drilling fluids are commonly preferred for drilling shale formations due to their strong shale inhibition properties which help maintain wellbore stability [11]. However, their widespread application is constrained by high costs and environmental risks. Water-based drilling fluids, being relatively cost-effective and environmentally less impactful, emerge as potential alternatives to oil-based drilling fluids [12]. Various additives, such as shale inhibitors, are introduced into water-based drilling fluids to enhance their shale hydration inhibition properties [13,14,15]. Potassium chloride (KCl) is a frequently used shale inhibitor in oil fields, having been employed for many years due to its effectiveness in reducing clay hydration. Nevertheless, it often requires high concentrations to exhibit its effectiveness, posing challenges in meeting the demands of complex drilling conditions. Additionally, in the event of the gradual depletion of salt, clay will undergo rehydration and expansion, exacerbating the instability of boreholes. Cationic polymers can adsorb onto the surface of shale, preventing its dispersion and further enhancing the shale inhibition of the KCl mud system, which is of concern in oilfield operations [16,17]. Correspondingly, researchers have developed high molecular weight partially hydrolyzed polyacrylamide (HPAM) to inhibit the hydration effects of clay. HPAM has the advantage of adsorbing on the clay surface and binding to multiple sites [18,19,20]. These actions effectively alleviate the dispersion of shale. Nevertheless, due to its large molecular weight, it cannot penetrate the interlayers of clay, thus limiting its ultimate effectiveness in inhibiting shale hydration. Another drawback is the insufficient coverage of the shale surface by HPAM, resulting in a lower capability for pore plugging, making this system less effective in blocking nanoscale pores. Recently, new shale inhibitors have been introduced into water-based drilling fluids, including surfactants, ionic liquids, amines, nanoparticles, and others [21,22,23,24]. Surfactants, while having strong foaming capabilities, face limitations in their widespread adoption [25]. Ionic liquids exhibit robust hydration inhibition, but their high cost hinders large-scale applications [26]. Amines, due to the adsorption of amine groups on shale surfaces through electrostatic forces and hydrogen bonding, demonstrate strong hydration inhibition and have garnered significant attention [27,28,29]. However, they have still not demonstrated any effectiveness in shale plugging.

Shale formations contain numerous micro and nano-scale pores and cracks. Considering the size compatibility, there has been significant interest in nanoscale plugging agents to address wellbore instability issues [30,31,32]. Traditional plugging materials, due to their larger size, struggle to form mud cakes to mitigate water invasion. In this regard, the advantages of nanoparticles become apparent, as they can effectively address these challenges. Among this class of materials, nano-silica (NS) has garnered the most research attention due to its high thermal stability, strong network bonding, excellent interface adhesion, and low toxicity [33,34]. NS is a hydrophilic material with the ability to adsorb water, enhancing the rheological properties and filtration control of water-based drilling fluids [35,36]. Most importantly, it possesses dimensions matching shale pores and cracks, enabling it to reduce water infiltration through plugging, showcasing promising application potential [37,38,39]. However, unmodified NS often aggregates, limiting the performance; functionalization becomes an effective method to enhance its overall capabilities. Considering the potent inhibition nature of amines, the functionalization of NS with amine groups may yield a novel high-performance shale stabilizer with both plugging and inhibition properties.

(3-Aminopropyl) triethoxysilane (APTES) containing amine groups has been confirmed to enhance the dispersion stability of NS in aqueous solutions. Both APTES and NS are mature industrial products with low production costs. Silane-coupling modification is also a straightforward and cost-effective functionalization method [40,41]. Therefore, the grafting of APTES onto the surface of NS may create a novel shale stabilizer (ANS) that is easily amenable to industrial-scale production. This lays a solid foundation for its potential field application. In this study, ANS was prepared using the silane-coupling method. The structure of ANS was characterized through Fourier transforms infrared spectroscopy (FT-IR), thermo-gravimetric analysis (TGA), and particle size tests (PST). The shale stabilizing properties of ANS were evaluated through tests such as pressure penetration, BET analysis, hydration expansion and dispersion. Furthermore, the interaction between ANS as a shale stabilizer and clay was explored through clay zeta potential and particle size analysis. Additionally, the shale stabilizing mechanism of ANS was comprehensively analyzed.

## 2. Materials and Methods

### 2.1. Materials

NS (99.8%), APTES (99%), ethanol (99.7%), ammonia (25–28%), KCl (99%) were purchased from the Aladdin Reagent Company (Shanghai, China). Sodium bentonite was provided by Yuancheng bentonite Company (Weifang, China). The shale sample used in this study was water-sensitive shale, and its mineral composition included quartz (42%), potassium feldspar (7%), plagioclase (15%), calcite (17%), and clay (19%).

### 2.2. Preparation of ANS

**Figure 1:**
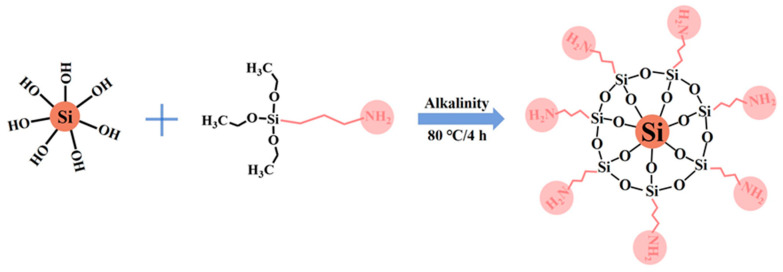
Preparation process of ANS.

The preparation of ANS was carried out using the process mentioned in Figure 1. Initially, 5 g of NS was uniformly dispersed in a mixed solution of ethanol and water through ultrasonic treatment. Then, 2.5 g of APTES was added to the above mixture, and its pH was adjusted to alkaline using ammonia. The aminated nano-silica, denoted as ANS, was obtained through a reaction at 80 °C for 4 h. 

### 2.3. Characterization of ANS

The Nicolet iS5 spectrometer (Thermo Fisher Scientific, Waltham, MA, USA) was utilized to observe the FT-IR spectra of ANS in the wavenumber range of 400 to 4000 cm^−1^. The TG 209 F1 instrument (NETZSCH Analyzing & Testing, Selb, Germany) was employed to measure the thermal weight loss of ANS, covering a temperature range of 30–600 °C with a heating rate of 10 °C/min under a nitrogen atmosphere. The thermal stability of ANS was analyzed based on the weight reduction during the process. The NanoBrook 90Plus instrument (Brookhaven Instruments, Holtsville, NY, USA) was employed to determine the particle size distribution of ANS, assessing whether its particle size is maintained within the micro-nano scale.

### 2.4. Pressure Penetration Test

Due to the extremely low permeability of shale, when exposed to water-based drilling fluids, the liquid gradually infiltrates the interior of the shale under the action of pressure differentials, increasing the pore pressure near the wellbore. This eventually results in the equilibration of the fluid column pressure with the formation pore pressure, leading to the loss of effective support for the wellbore by the drilling fluids. Additionally, the entry of a small amount of water into the shale may significantly alter the formation pressure, and changes in pore pressure can induce alterations in the effective stress state near the wellbore, thereby affecting wellbore stability. The interaction between drilling fluids and shale can be simulated through a pressure penetration test. It is a crucial method for evaluating the plugging effectiveness of shale stabilizers [42,43]. The impact of ANS on delaying fluid intrusion into rocks was evaluated using a pressure penetration experimental apparatus. Shale samples were processed into cylinders with a length of 6 mm and a diameter of 2.5 cm. The steps for the pressure penetration experiment were as follows: firstly, the shale cores were fully saturated with a 20% NaCl aqueous solution. Subsequently, upstream and downstream pressures were set at 2 MPa and 1 MPa, respectively, with a confining pressure of 5 MPa. Following this, the upstream fluid was replaced with different test fluids. Finally, while maintaining constant upstream and confining pressures, the experiment was initiated and changes in downstream pressure were recorded. It should be noted that all percentage concentrations in the article were based on the mass ratio of materials to deionized water.

### 2.5. BET Test

The hydration of shale can lead to changes in its pore structure. When nanofluids interact with shale, particle filling significantly reduces the surface area and pore volume of the shale. Therefore, the alteration in the microscopic structure of shale can reflect the plugging effect of the test liquids. The Brunauer–Emmett–Teller (BET) test is a commonly used method for characterizing material surface properties, primarily employed to assess the surface area and pore structure of materials [44]. Based on gas adsorption, the BET test measures the amount of gas adsorbed on the material surface to determine its specific surface area and pore structure. This method has been widely applied in the microscopic characterization of shale structures [45,46]. Shale samples were crushed to a particle size of 40 to 60 mesh. Subsequently, they were immersed separately in deionized water, 2% NS, and 2% ANS for 6 h. Afterward, the samples were retrieved, allowed to dry, and subjected to nitrogen adsorption experiments using the Quadrasorb SI instrument. Based on the nitrogen adsorption data, the surface area and pore volume of shale could be obtained through BET analysis [47].

### 2.6. Expansion Inhibition Test

When clay comes into contact with water-based drilling fluids, it undergoes expansion due to hydration. Therefore, the extent of clay expansion serves as a crucial indicator reflecting its degree of hydration. The NP-01 expansion apparatus is a precise device used to directly assess the expansion behavior of bentonite by measuring changes in the sample’s height. In this test, bentonite cores were prepared by compressing 10 g of bentonite at 10 MPa for 5 min in a mold. Subsequently, the cores were immersed in various test liquids for 24 h. The changes in sample height were recorded to reflect the inhibitory effect of the shale stabilizer.

### 2.7. Dispersion Inhibition Test

When shale comes into contact with water-based drilling fluids, it undergoes dispersion due to hydration. Therefore, the degree of shale dispersion serves as a crucial indicator, reflecting its degree of hydration. Particularly, hydration tends to be more intense at high temperatures, leading to increased dispersion. Hence, it is essential to assess the dispersion of shale at elevated temperatures in the test liquids. In this test, shale samples were crushed to a particle size of 6–10 mesh for use in this experiment; 50 g of shale fragments were weighed and added to 350 mL of different test liquids. They were transferred to an aging tank and heated at 120 °C for 16 h. After aging, a 40-mesh sieve was used to screen the shale fragments. The remaining fragments were weighed after drying at 105 °C. The ratio of this weight to the initial weight represented the rolling recovery rate.

### 2.8. Clay Zeta Potential and Particle Size Test

Shale stabilizers often interact with clay, leading to changes in the clay’s zeta potential and particle size distribution [48]. Therefore, it is necessary to investigate these aspects to elucidate the inhibition hydration mechanism of ANS. In this experiment, 2 g of bentonite was added to 100 mL of deionized water and stirred for 24 h to ensure complete hydration. Subsequently, different concentrations of ANS were added, and stirring continued for 12 h. The zeta potential of bentonite was measured using the NanoBrook 90Plus instrument to analyze the impact of ANS on its zeta potential. The particle size distribution of bentonite was determined using the Bettersize 2600 instrument (Bettersize Instruments, Dandong, China) to assess the impact of ANS on its particle size.

## 3. Results

### 3.1. Characterization of ANS

#### 3.1.1. FT-IR

**Figure 2:**
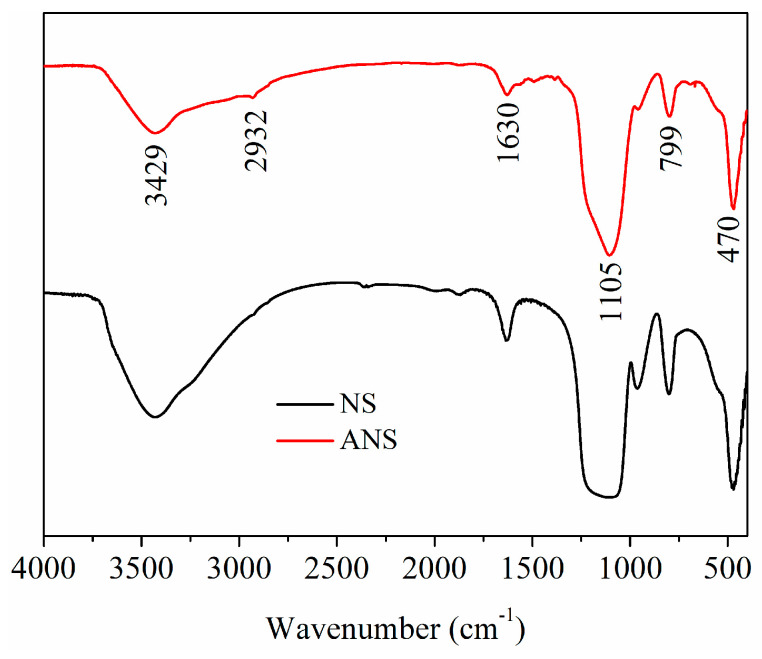
Fourier transforms infrared spectroscopy curves of NS and ANS.

Figure 2 presents the FT-IR curves of NS and ANS. The Si-OH vibration near 1630 cm^−1^, and the Si-O-Si vibration peaks at 799 cm^−1^, 470 cm^−1^ and 1105 cm^−1^ were reflected in the curves [49]. Clearly, ANS retained the characteristic peaks of the silica framework. In comparison, the O-H absorption peak around 3429 cm^−1^ significantly weakened, while a stretching vibration absorption peak of C-H bonds appeared at 2932 cm^−1^. This suggested that the silane-coupling agent APTES had reacted the Si-OH groups [50], successfully bonding to the surface of NS particles.

#### 3.1.2. TGA

**Figure 3:**
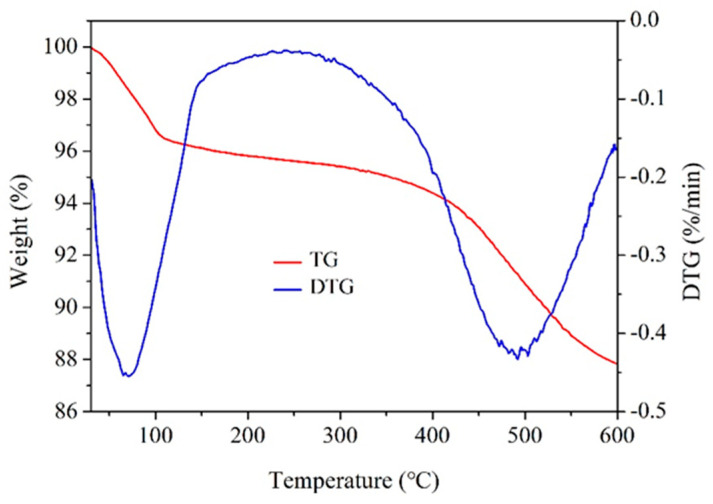
Thermo-gravimetric and Derivative Thermo-gravimetric curves of ANS.

Due to the commonly encountered high-temperature conditions in geological formations, the drilling fluids must withstand elevated temperatures. Otherwise, they cannot perform effectively. Therefore, it is essential to investigate the thermal stability of ANS. Figure 3 illustrates the TG and DTG curves of ANS. In the initial stage, there was approximately a 4% weight loss below 120 °C, attributed to solvent evaporation. The weight only slightly decreased as the temperature increased to the 120–400 °C range. Around 400 °C, the TG curve sharply declined, indicating the thermal degradation of functional groups on the silica surface. In addition, the DTG curve also confirmed that there was significant thermal decomposition due to solvent evaporation in the first stage, but the maximum thermal decomposition occurred around 490 °C. Therefore, ANS exhibited excellent thermal stability, a crucial property for water-based drilling fluids that needed to withstand high temperatures during the drilling process. 

#### 3.1.3. PST

**Figure 4:**
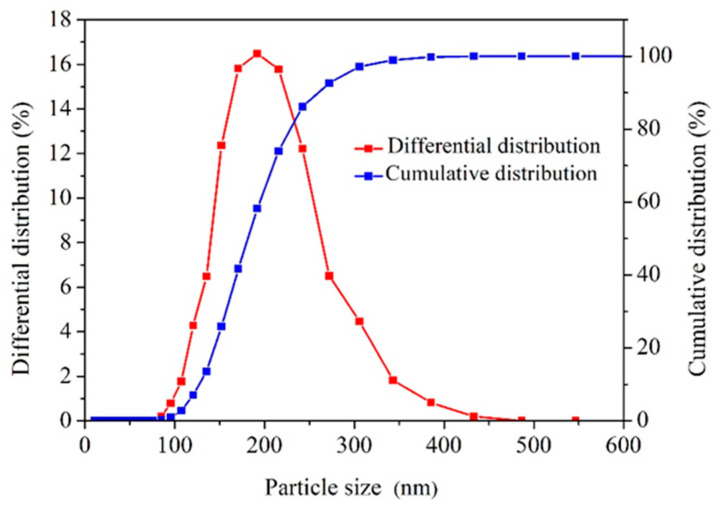
Particle size distribution curves of ANS.

Shale belongs to dense rocks with unique pore structure characteristics. The optimal performance of the dense plugging is achieved when the particle size of the shale stabilizers matches the pore dimensions of the shale. Therefore, it is necessary to investigate whether the particle size distribution of ANS corresponds to the micro-nano pore sizes in shale. Figure 4 depicts the PST curves of ANS. The average size of ANS was 183.58 nm, with a particle size distribution ranging from 85.01 to 433.11 nm. This size range corresponded well with the dimensions of shale pores and cracks, facilitating efficient plugging.

### 3.2. Pressure Penetration

**Figure 5:**
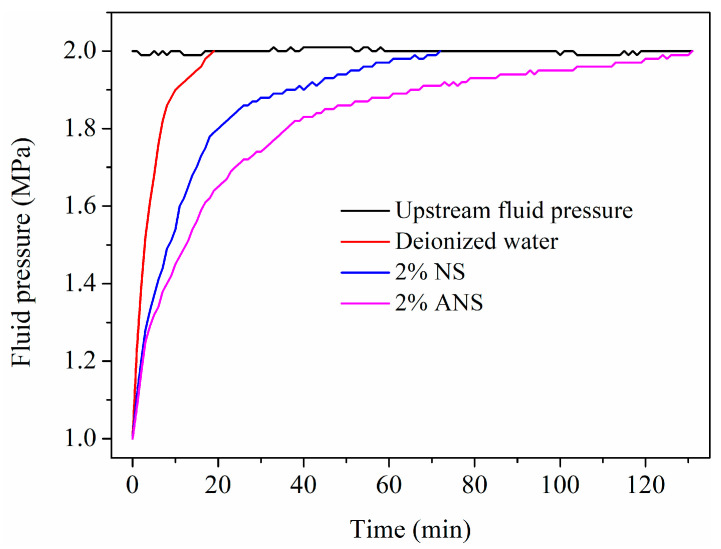
Pressure penetration curves for different test fluids.

Nanoparticles are widely employed in shale plugging due to their size compatibility with shale. Pressure penetration tests can simulate the process of nanoparticles invading shale pores and cracks [51]. Therefore, this method has become a crucial approach for studying the effectiveness of nano-sized plugging agents. The test fluids can be pumped into the shale through upstream pressure. If they exhibit strong plugging performance, the pressure penetration rate will be slow, requiring more time for downstream pressure to balance with upstream pressure. If their effectiveness is limited, a plugging structure will not form on the shale surface, leading to a rapid pressure penetration rate. Accordingly, the pressure equilibrium will be achieved in a very short period of time. Figure 5 shows the pressure penetration curves for different test fluids. It reached an upstream pressure in 19 min within deionized water, indicating a rapid penetration into shale. The pores and cracks in the shale served as the main pathways for fluid flow. In the absence of any treatment agents, fluids could easily penetrate into the subsurface through these channels. Therefore, under conditions with a pressure differential, downstream pressure increased rapidly. Pressure equilibrium was achieved in a very short period given the ease of fluid movement along these pathways. However, after adding nanoparticles, the pressure penetration rates were significantly slowed down. For NS and ANS, it took 72 and 131 min, respectively, to reach upstream pressure. Due to the size compatibility of nanoparticles with shale pores, they could penetrate the interior of shale, densely accumulate, and form a plugging layer. Consequently, in this test, nanoparticles exhibited a pronounced plugging effect. For NS, the pressure penetration equilibrium time was 3.79 times longer than that of the control sample. Surprisingly, for ANS it increased to 6.89 times. Particularly, ANS demonstrated stronger plugging performance, mainly attributed to the modification of the silica surface using APTES, allowing the amine groups to adsorb more tightly on the shale surface, resulting in a denser plugging layer [52]. It was more effective in retarding pore pressure penetration. This confirmed that ANS was a shale stabilizer with stronger plugging performance, exhibiting a significant effect in impeding pore pressure penetration.

### 3.3. BET Investigation

**Figure 6:**
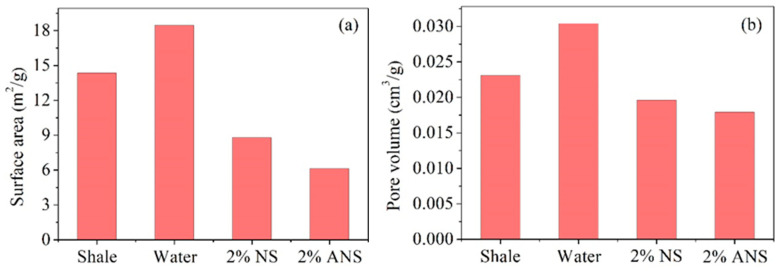
Test results of (**a**) surface area and (**b**) pore volume of shale after immersion in different test fluids.

The changes in the microstructure of shale after the exposure to various test fluids are crucial indicators of its plugging performance. Figure 6 shows the test results of surface area and pore volume of shale after immersion in different test fluids. The initial surface area of the shale was 14.36 m^2^/g. After immersion in deionized water, it increased to 18.45 m^2^/g. The clay particles on the surface of shale exhibited a high affinity for adsorbing water. Upon contact with water-based fluids, the clay surfaces adsorbed a significant amount of water, generating substantial swelling pressure that led to the expansion of fractures. Clearly, the increase in surface area after exposure to water was mainly attributed to the hydration effect, resulting in wider pores and cracks [53,54]. Subsequently, after the immersion in NS and ANS, the surface area decreased to 8.79 and 6.12 m^2^/g, respectively. This indicated that both NS and ANS could enter shale pores and cracks to play a plugging role. On one hand, nanoparticles could penetrate shale pores and cracks, forming plugging structures that reduce water infiltration. Consequently, the expansion pressure generated by hydration was diminished. On the other hand, nanoparticles could adsorb onto clay surfaces, contributing significantly to the reduction in surface area. Notably, with ANS, a greater reduction in surface area was observed, confirming its enhanced adsorption capacity, thereby favoring the formation of a denser plugging layer. The results of the shale pore volume test corresponded well with the surface area test results. After the immersion in deionized water, the pore volume increased from 0.02308 to 0.03039 cm^3^/g. The hydration effect resulted in wider pores and fractures, corresponding to an increased pore volume. However, after the immersion in NS and ANS, it decreased to 0.01961 and 0.01792 cm^3^/g, respectively. The entry of nanoparticles into shale pores and cracks evidently reduced their pore volume. A lower pore volume implied enhanced plugging capability. This further confirmed that ANS had a stronger plugging effect. This was mainly due to the strong adsorption of amine groups in ANS, forming a dense plugging layer, significantly reducing the size of shale pores and cracks. This dense plugging effectively blocked water intrusion, and the adsorption on the shale surface further increased the difficulty of water adsorbing onto the clay surface, thereby effectively inhibiting shale hydration. 

### 3.4. Expansion Inhibition

**Figure 7:**
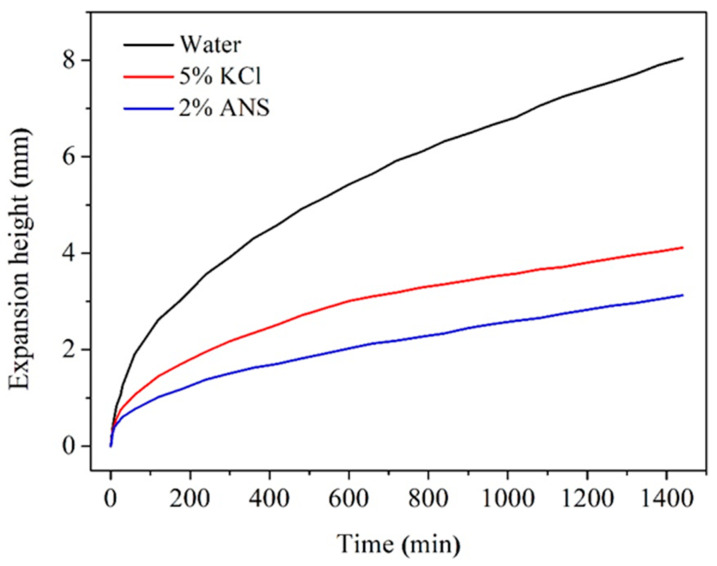
Test results of the expansion height for bentonite in different test fluids.

Shale is rich in clay minerals, and all types of clay minerals exhibit a strong affinity for water. Among them, montmorillonite, a type of layered clay mineral, possesses the greatest natural attraction to water due to the continuous expansion of its lattice structure. Bentonite, characterized by its abundant content of this mineral and low cost, is widely available. Therefore, it can be chosen as a suitable material for evaluating the hydration behavior of clay. The expansion inhibition test can reflect the ability of inhibitors to prevent clay hydration expansion. As shown in Figure 7, the test results of the expansion height for bentonite in different test fluids are presented. Specifically, the expansion height in deionized water increased rapidly with time, reaching 8.04 mm within 24 h. This implied that in deionized water, clay underwent thorough hydration. Therefore, this aspect should not be overlooked in studies on shale stability. In contrast, the reduction in expansion height was attributed to the effective inhibition of two inhibitors. The expansion heights decreased to 4.12 mm in 5% KCl and 3.13 mm in 2% ANS, respectively. With a smaller dosage, ANS achieved a lower expansion height compared to KCl. It was evident that ANS exhibited stronger hydration inhibition than commonly used KCl. This was due to its micro-nano dimensions, allowing it to plug shale pores and cracks, thereby reducing water infiltration into the shale. In addition, grafting APTES onto the surface of silica could offer prolonged clay stability. This was attributed to the abundance of amino groups, which could strongly electrostatically adsorb onto the clay surface. Consequently, only a reduced amount of water could penetrate the clay, achieving effective clay stabilization. The above analysis indicated that ANS demonstrated significant potential as a shale inhibitor. 

### 3.5. Dispersion Inhibition

**Figure 8:**
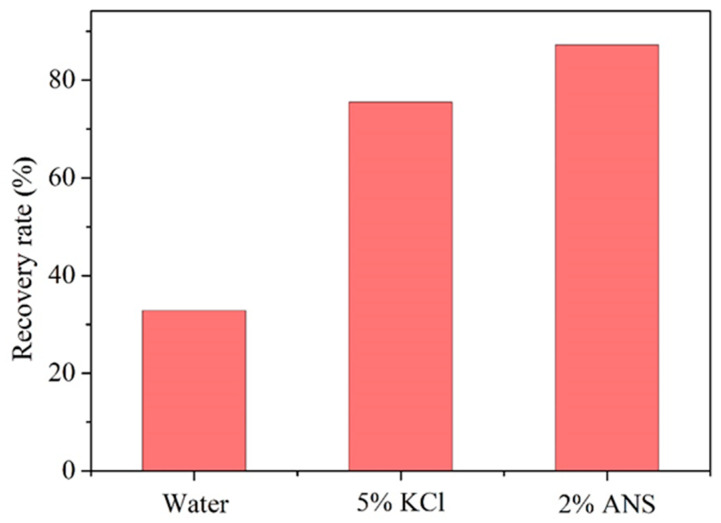
Test results of the rolling recovery rate for shale in different test fluids.

Shale, rich in clay minerals, exhibits strong hydrophilicity. Upon contact with water-based fluids, it easily weakens the bonding forces between shale particles, leading to shale dispersion and a subsequent reduction in strength, which is detrimental to wellbore stability. Additionally, since formations are often in high-temperature environments, the dispersion of shale under elevated temperatures becomes a focal point in studying its stability. A dispersion inhibition test is conducted to simulate the dispersion behavior of shale during drilling. Figure 8 illustrates the test results of the rolling recovery rate for shale in different test fluids. Specifically, the recovery rate in deionized water was very low, at only 32.84%. This result also confirmed the pronounced dispersibility of shale in water-based fluids. In contrast, the increase in the rolling recovery rate was attributed to the effective inhibition of two inhibitors. The recovery rates increased to 75.49% in 5% KCl and 87.22% in 2% ANS, respectively. With a smaller dosage, ANS achieved a higher shale recovery rate compared to KCl. This indicated that silica modified with APTES effectively controlled the dispersion of shale cuttings. It could be inferred that, due to the higher cationic density, the amine cations presented in ANS adsorbed more strongly to the shale surface. This resulted in a firm fixation of shale cuttings during the shale rolling recovery test, ensuring that shale cuttings remained intact. The test confirmed that the newly prepared ANS achieved a higher shale recovery rate compared to the commonly used KCl, suggesting its stronger inhibition properties that impeded the dispersion of shale cuttings. Therefore, ANS presented itself as a shale stabilizer with significant application potential.

### 3.6. Clay Zeta Potential and Particle Size

**Figure 9:**
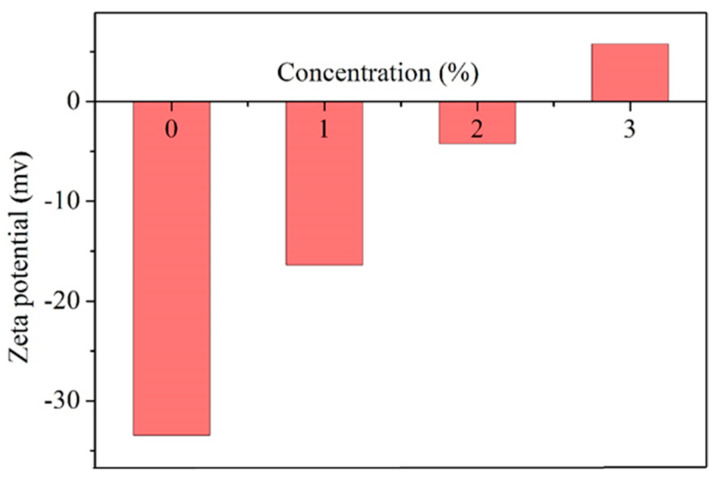
Zeta potential of bentonite treated with different test fluids.

The interaction between shale inhibitors and clay typically affects the zeta potential of bentonite particles in aqueous dispersion. Therefore, it is necessary to investigate the impact of inhibitors on the electrokinetic properties of bentonite particles. The zeta potential of bentonite treated with different test fluids is illustrated in Figure 9. In deionized water, the zeta potential was −33.47 mV, indicating the complete dispersion of bentonite particles. The adsorption of ANS at different concentrations would impact the zeta potential of particles to varying degrees. As the concentration of ANS increased, its absolute value continued to decrease. This phenomenon was attributed to the interaction between the amine groups in ANS and the negative charges in bentonite [55]. In particular, when the ANS concentration reached 3%, the zeta potential changed from a negative value to a positive value, indicating a strong interaction with the clay [56]. The surface of silica modified with APTES was rich in amino groups. These amino groups underwent protonation in water, forming ammonium ions and thereby adsorbing onto negatively charged sites on clay through electrostatic attraction. Additionally, hydrogen bonds typically existed between amino groups and clay, further contributing to the reduction in the zeta potential [57,58]. In summary, ANS could strongly adsorb on the clay surface through electrostatic and hydrogen bonding interactions, neutralizing its negative charge, and therefore, reducing its hydration effect [59]. When the potential of bentonite particles decreased by 20%, typically, their hydration and expansion would be thoroughly suppressed. Therefore, the adsorption of ANS significantly weakened the tendency of clay hydration, consistent with the results of bentonite expansion and shale dispersion tests. 

**Figure 10:**
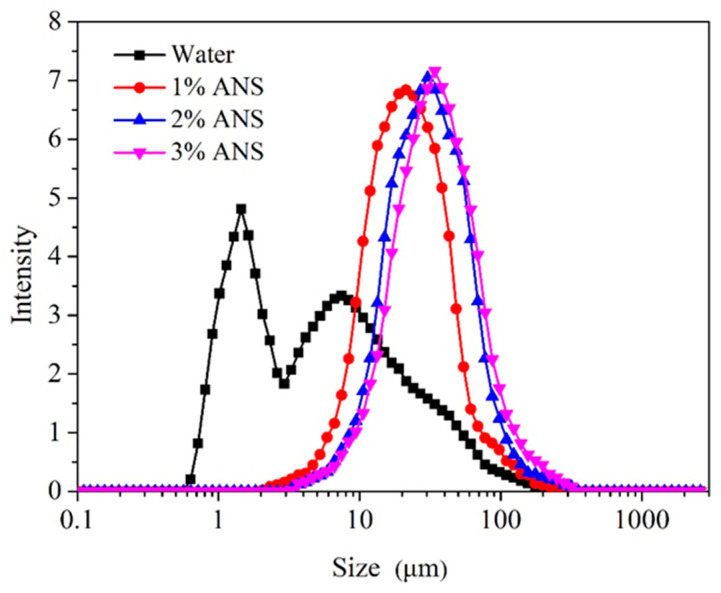
Particle size of bentonite treated with different test fluids.

The particle size of clay particles to some extent reflects their hydration and dispersion state. Therefore, testing the impact of inhibitors on the particle size of clay particles can reflect their inhibition mechanism. The particle size of bentonite treated with different test fluids is depicted in Figure 10. In deionized water, the particle size was 4.89 μm. As the concentration of ANS increased, the average particle size continuously increased, shifting the particle size curves to the right. When the ANS concentration reached 3%, the particle size of bentonite had reached 32.27 μm. ANS nanoparticles adsorbed on the clay surface through amine groups [60]. The more ANS added implied more clay aggregation, leading to an increase in particle size [61]. This result was in good agreement with the zeta potential test result, confirming the hydration dispersion inhibition capacity of ANS. It also explained the mechanism behind the inhibition of clay hydration expansion and dispersion.

### 3.7. Wellbore Stability Mechanism Analysis

**Figure 11:**
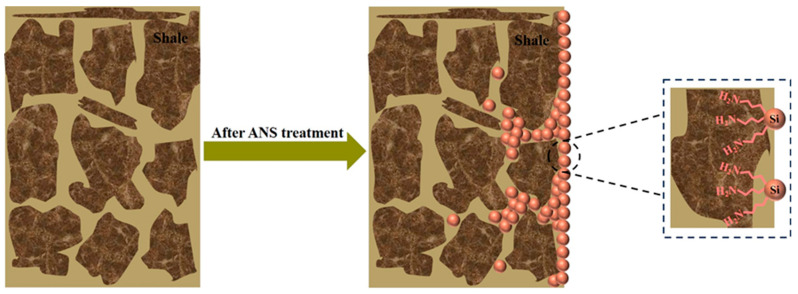
Schematic diagram of the wellbore stability mechanism for ANS.

Based on the above study, the wellbore stability mechanism of ANS as a novel shale stabilizer is summarized in Figure 11. Firstly, ANS had micro-nano dimensions, allowing it to enter the pores and cracks of shale, thereby forming a dense plugging layer. After treatment, the shale pressure penetration rate significantly slowed down, and the surface area and volume notably decreased. On the other hand, ANS contained numerous amine groups, which could adsorb on the shale surface through electrostatic and hydrogen bonding interactions, neutralizing the negative charge of shale. This would be advantageous in inhibiting the shale hydration expansion and dispersion. In conclusion, ANS played a dual role in dense plugging and hydration inhibiting, making it a novel shale stabilizer with potential applications in drilling. 

## 4. Conclusions

A novel shale stabilizer, ANS, was synthesized by introducing numerous amine groups onto the surface of nano-silica through silane-coupling modification. Its plugging performance was confirmed to be superior to nano-silica through pressure penetration and BET tests. ANS could increase the shale pressure penetration time from 19 to 131 min. Its hydration inhibition performance was demonstrated to be superior to the commonly used KCl through expansion inhibition and dispersion inhibition tests. ANS was able to reduce the expansion height of bentonite from 8.04 to 3.13 mm and increase the shale recovery rate from 32.84% to 87.22%. Firstly, ANS possessed micro-nano dimensions, allowing it to enter the pores and cracks of shale, thereby forming a dense plugging layer. Secondly, ANS contained numerous amine groups that could adsorb on the shale surface through electrostatic and hydrogen bonding interactions, neutralizing the negative charge of shale and inhibiting its hydration expansion and dispersion. Therefore, ANS exhibited a dual role in providing dense plugging and effective hydration inhibition, making it a promising novel shale stabilizer during the drilling process. In the future, the compatibility between ANS and drilling fluids will be evaluated, with a focus on their dense plugging and hydration inhibition when used in drilling fluids. This evaluation will help to further optimize the drilling fluid formulation, enabling ANS to be put into field application as early as possible.

## Data Availability

Data are contained within the article.

## Abbreviations

ANS	Aminated nano-silica
APTES	(3-Aminopropyl) triethoxysilane
FT-IR	Fourier transforms infrared spectroscopy
TGA	Thermo-gravimetric analysis
PST	Particle size tests
KCl	Potassium chloride
HPAM	Partially hydrolyzed polyacrylamide
NS	Nano-silica
BET	Brunauer-Emmett-Teller
